# 12-month follow-up of intensive outpatient treatment for PTSD combining prolonged exposure therapy, EMDR and physical activity

**DOI:** 10.1186/s12888-024-05656-9

**Published:** 2024-03-26

**Authors:** Julie Rendum Klaeth, Andreas Gjerde Jensen, Trude Julie Brynhildsvoll Auren, Stian Solem

**Affiliations:** 1 Regional Unit for Treatment of Severe Posttraumatic Stress Disorder, Nidaros DPS, St. Olavs Hospital, Trondheim, 7040 Norway; 2 Department of Psychology, Norwegian University of Science and Technology, Trondheim, 7491 Norway

**Keywords:** Brief, Concentrated, EMDR, Intensive, Long-term follow-up, Massed, PE, Physical activity, PTSD, trauma

## Abstract

**Background:**

Preliminary evidence shows promising treatment outcomes at short-term follow-up for intensive posttraumatic stress disorder (PTSD) treatment, but long-term follow-up studies are sparse. This study is a sequel to a previous pilot study and open trial, set out to investigate treatment outcomes at 12-month follow-up for outpatients completing an 8-day intensive treatment for PTSD.

**Methods:**

All patients were diagnosed with PTSD and had multiple previous psychotherapy attempts (*M* = 3.1). Patients were assessed at pre-treatment, post-treatment, 3- and 12-month follow-up. Of 35 treated patients, 32 (91.4%) attended the long-term follow-up assessment. The treatment programme combined prolonged exposure therapy, eye movement desensitization and reprocessing, and physical activity.

**Results:**

The effect sizes indicated large reductions in symptoms of PTSD, depression, anxiety, interpersonal problems, and well-being. Changes in functioning showed a small-medium effect. Results were stable across the follow-up period. The treatment response rates showed that 46–60% of patients achieved recovery with respect to PTSD symptoms, and that 44–48% no longer met diagnostic criteria for PTSD.

**Conclusions:**

Time-limited and concentrated outpatient treatment for PTSD can yield large and enduring positive outcomes. Controlled trials are needed to establish relative efficacy.

**Trial registration:**

The study was registered in Current Research Information System In Norway (Cristin). Cristin-project-ID: 654,790. Date of registration: 18.03.2019.

## Background

Intensified session frequency in treatment of post-traumatic stress disorder (PTSD) has been linked with enhanced engagement and motivation through experience of early gains, less distractions from the therapeutic process, and less avoidance [1, 2]. High session frequency in outpatient treatment for PTSD has also been associated with greater symptom reduction. A study investigating the impact of session frequency on treatment outcome (average days between sessions ranged from 2 to 32) found that higher average days between sessions was associated with less reduction of PTSD symptoms [3]. Intensive outpatient programmes (IOPs) show equivalent treatment effect and less dropout compared with standard outpatient treatment (commonly defined as maximum 2 sessions per week) [4, 5]. However, the evidence for both intensive and traditional weekly therapy for PTSD largely relies on short-term outcomes, as there is a dearth of research on long-term follow up (LTFU).

A meta-analysis using randomized controlled trials of non-intensive psychological treatments for PTSD with LTFU (i.e. minimum 12 months after treatment completion) found 22 eligible studies [6]. They concluded that there only existed solid research on LTFU for cognitive behavioural therapies (CBT) for PTSD with an effect size of 1.36. One of the studies included in the meta-analysis examined LTFU up to 10 years after treatment [7]. At 9-month follow-up, 50–55% did not meet the criteria for PTSD [8], and the sample improved further during the LTFU (5–10 years) as 78–82% no longer suffered from PTSD [7]. The improvement at LTFU was not explained by further treatment in the follow-up period. In fact, additional treatment was actually associated with worse outcome at LTFU.

Regarding LTFU of IOPs there exists less research. One study on 3-week cognitive processing therapy reported large changes from pre-treatment to 12-month follow-up (PTSD; *d* = 1.28, and depression; *d* = 1.18) [9]. Another study on 2-week PE (prolonged exposure therapy) showed large reductions in PTSD (*d* = 0.88) and depression (*d* = 0.81) at 12-month follow-up [10]. A third study on 10-day EMDR (eye movement desensitization and reprocessing) treatment reported significant symptom reduction and large effect sizes at 12-month follow-up [11]. These studies indicate that gains achieved from IOPs for PTSD persists. However, the response rates were low in the first two studies (37% and 52%) [9, 10], and the third study only had a sample size of 15 [11].

At our public PTSD clinic situated in the city of Trondheim, Norway, we have implemented an IOP [12] based on a Dutch inpatient programme [13]. The Dutch programme consisted of an 8-day intensive treatment combining individual PE and EMDR, psychoeducation, physical activity (PA), and therapist rotation. The Dutch study reported large effect sizes on reduction in PTSD symptoms from pre- to post-treatment. From post-treatment to 6-month follow-up the results showed a small, but significant, increase in PTSD symptoms (based on clinical interview). This increase was not evident in patients’ self-reported symptoms. The effect sizes remained large from pre-treatment to 6-month follow-up (*d* = 1.70–1.99). Furthermore, 91.7% of the patients showed a clinically meaningful treatment response with at least 10 points reduction on the Clinician Administered PTSD Scale for DSM-5 (CAPS-5), and 67% lost their PTSD diagnosis. LTFU data has not been reported. In our implementation of the Dutch programme as an outpatient programme, all the treatment elements were kept, but time allocated to group physical activity and psychoeducation was reduced. The programme yielded significant improvement in PTSD symptoms at post-treatment and 3-month follow-up (*d* = 1.38–1.52) [14]. Patients also reported significant improvement in their scores on depression, anxiety, well-being, and interpersonal functioning. There was no dropout and the treatment satisfaction was high [14].

As there is a lack of long-term follow-up studies on the effectiveness of intensive treatment for PTSD, this study will report outcomes at 12-month follow-up for patients included in a previous pilot study [12] and an open trial [14]. The current study aimed to explore the maintenance of gains 12 months after receiving an 8-day IOP, in a sample of previous treatment non-responders. Based on the large effect sizes at 3-month follow-up in the pilot study [12] and the open trial [14], as well as the large effects observed at 6-month follow-up in the Dutch study [13], we hypothesized that gains would be maintained at 12-month follow-up.

## Methods

### Participants and procedure

This 12-month follow-up study consisted of data collected from patients participating in a pilot study [12] and an open trial [14] of an 8-day intensive treatment programme for PTSD. All patients referred had to be 18 years or older, previously diagnosed with PTSD, and had to have at least one previous treatment attempt for their PTSD. Exclusion criteria included acute suicidality, psychosis, severe drug addiction, and insufficient fluency in Norwegian. Prior to inclusion, patients underwent a diagnostic assessment, including medical and mental health history, and completed relevant self-report questionnaires. Diagnosis was determined by using diagnostic interviews, either the MINI Plus 5.0 interview [15] or the ADIS-IV [16], in addition to the PTSD Symptom Scale Interview (PSS-I) [17]. Of the patients referred to the clinic between August 2018 and March 2021, 31.8% chose intensive treatment over traditional outpatient treatment. Five of these were not eligible to partake in the study due to either not consenting to research, not showing up at treatment start, or treatment deviating from the protocol (i.e. group size less than three). This left 35 eligible participants for analysis.

In the interim between assessment and the 8-day intensive programme, the patients had 3.2 (*SD* = 0.9) preparatory sessions. The preparation included defining targets for imaginal exposure, constructing in vivo exposure tasks, and receiving further information about the programme. The eight days had a similar structure. Each day consisted of 90 min individual PE, 45 min group physical activity (PA), 90 min individual EMDR, and 45 min group psychoeducation, in that order. The PE sessions followed the PE protocol including imaginal exposure, processing, and homework assignments [18]. The participants listened to audio recordings of the imaginal exposure and completed in vivo exercises at home daily. In the EMDR sessions, the focus was on desensitization, installation, body scan, and closure; targeting trauma memories, triggers and future templates, following the EMDR protocol [19].

To prevent therapist drift and under-utilization of exposure interventions, therapists rotated between patients from session to session. Patients met between four to seven different therapists during the programme, depending on the size of the patient group. The psychoeducation took place in a group setting (three to six patients) and focused on themes from the PE manual; posttraumatic symptoms as normal reactions to trauma, the rationale for exposure treatment, avoidance, negative thoughts, feelings, self-esteem, and relapse prevention. The PA, led by a physiotherapist, was also group based. The goal of the PA was to give the patients exercises of moderate intensity, that demanded attention and activation of the whole body, and to give them experiences of mastery and strength. All participants were offered an individual follow-up session at two weeks, three months, and 12 months after the intensive programme.

The sample had a mean age of 38.5 years, and most participants were female (91.4%). Twenty-nine patients had Norwegian as their first language and all participants spoke Norwegian adequately. The sample had 3.1 (*SD* = 1.6, range 1–6) prior psychotherapies, defined as evenly sessions attended for at least three months. Traumas reported included domestic violence, childhood sexual and physical abuse, rape, terrorist attack, witnessing murder, and war. Most of the patients had experienced multiple traumas (*n* = 31, 88.6%) and 25 (71.4%) reported sexual traumas. The mean number of years since symptoms of PTSD first occurred was 15.8 years (*SD* = 10.8). 51% (*n* = 18) used current psychotropic medication including benzodiazepines (*n* = 9), antidepressants (*n* = 7), antipsychotic (*n* = 4), and stimulants (*n* = 1). The nine patients on prescription benzodiazepines were encouraged to abstain from use during treatment, which they adhered to. Other medications were stable during treatment and there were no reports on changes in medications during follow-up. Twelve patients had one comorbid disorder, one patient had two comorbid disorders, two had three comorbid disorders, while 19 were not diagnosed with a comorbid disorder. Comorbid disorders included: Attention deficit/hyperactivity disorder (*n* = 2), social anxiety disorder (*n* = 2), depression (*n* = 11), personality disorder (*n* = 3), obsessive-compulsive disorder (*n* = 1), alcohol abuse (*n* = 1), and unspecified eating disorder (*n* = 1). A summary of the group´s background information is displayed in Table 1.

**Table 1:** Sample characteristics at pre-treatment

	M	SD	n	%
Age	38.46	12.58		
Female gender			32	91.4
Duration of disorder in years	15.81	10.96		
Previous psychotherapies	3.11	1.60		
Previous psychopharm. tx			26	74.3
Current psychopharm. tx			18	51.4
Comorbid disorder			16	45.7
Previous drug abuse			10	28.6
Current drug abuse			1	2.9
Employment				
Full-time work			4	11.4
Part-time work			2	5.7
Student			2	5.7
Sick leave			8	22.9
Work assessment allowance			10	28.6
Disability pension			8	22.9
No employment			1	2.9
Civil status				
Single			15	42.9
In a relationship			4	11.4
Cohabitant			8	22.9
Married			7	20.0
Divorced			1	2.9
Ethnic minority			6	17.1

*Note*. psychopharm. tx = psychopharmacological treatment

### Measures

A collection of self-report questionnaires and a structured diagnostic interview for PTSD were used to measure symptoms pre-treatment, two weeks post-treatment, and at 3- and 12-month follow-up. Independent assessors administered the diagnostic interviews conducted after treatment.

Diagnostic status was assessed using the PSS-I [17] at pre-treatment, post-treatment, and at 3- and 12-month follow-up. The PSS-I is a diagnostic interview corresponding with the PTSD criteria in DSM-IV. It consists of 17 items, scaled from 0 to 3 with a total score from 0 to 51. To meet diagnostic criteria, according to the PSS-I, the patient needs at least one endorsed symptom (score 1 or more) on “re-experiencing” (items 1–5), at least three from “avoidance” (items 6–12), and at least two from “arousal” (items 13–17). This study defined a cut-off score at 20 and a reliable change index (RCI) at 8 points. Cronbach’s alpha was 0.75. The updated PSS-I-5 was not available in Norwegian at the start of the study.

Self-reported PTSD symptoms were assessed using the PTSD Checklist for DSM-5 (PCL-5) [20]. The PCL-5 consists of 20 items on a scale from 0 to 4 (not at all to extremely), with a total score ranging from 0 to 80. To meet diagnostic criteria when using PCL-5 it is required with at least one endorsed symptom (score 2 or more) on “intrusion” (items 1–5) and “avoidance” (items 6–7), and two on “cognitions and mood” (items 8–14) and “arousal” (items 15–20). As in the Improving Access to Psychological Therapies Programme [21], this study used a cut-off at 32 and the RCI was set at 10 points. Cronbach’s alpha was 0.84.

To assess severity of depression and anxiety, this study used the Beck Depression inventory (BDI-II) [22] and the Beck Anxiety Inventory (BAI) [23]. Both scales consist of 21 items, rated from 0 to 3. Higher scores represent higher levels of symptoms. Grade of severity are typically divided in mild (14–19), moderate (20–28), and severe (29–63) for the BDI-II, and minimal (0–7), mild (8–15), moderate (16–25), and severe (26–63) for the BAI. Cut-off was in this study set to 11 with the RCI at 12 points for the BAI, and at 15 points on the BDI-II with a RCI of 9 points. Cronbach’s alpha for BDI was 0.86, and for BAI 0.91.

To assess the impact of the patients’ mental health on their functional capacity at home, in social activities, personal relationships, and at work, this study used the Work and Social Adjustment Scale (WSAS) [24]. WSAS has five items rated on a scale from 0 to 8 (not at all to very severely). Higher scores indicate lower functioning (< 15 mild, 15–30 moderate, and > 30 severe). The present study set the cut-off to 17 and the RCI to 8 points. Cronbach’s alpha was 0.77.

The patients’ subjective psychological well-being was assessed using the five-item World Health Organization Well-Being Index (WHO-5) [25]. Five positively phrased items are rated from 0 to 5 (none of the time to all of the time). Scores are multiplied, resulting in a total range from 0 to 100. In the present study, cut-off was set to 29 as previously done in a study on depression [26] and the RCI at 10 points. Cronbach’s alpha was 0.83.

Interpersonal distress was assessed with the Inventory of interpersonal problems (IIP-64) [27] consisting on 64 items, rated on a 0–4 scale. These results are presented as mean item scores. Higher levels on interpersonal problems result in higher scores. As commonly used in studies with Norwegian samples, a cut-off value of 1.03 and a RCI of at least 0.38 was used. Cronbach’s alpha was 0.89.

### Statistical analyses

Missing data was imputed using the expectation-maximization method [28]. Little’s missing completely at random test indicated that data was missing completely at random (*x*^2^ = 191.79, *p* = .85). Repeated measures ANOVA was used to test for changes in symptoms, functioning, interpersonal problems, and well-being. The analyses used data from four assessments (pre-treatment, post-treatment, and 3- and 12-month follow-up). Effect sizes were reported using partial eta squared (*η*^2^) and Cohen’s *d* (using pooled *SD*). Typical standards for *η*^2^ are small (0.01), medium (0.06), and large (0.14), while for Cohen’s *d* the most common cut-offs are small (0.2), medium (0.5) and large (0.8). Treatment response rates were also calculated using percentages scoring below clinical cut-off, achieving reliable change, and clinically significant change (recovery) which combines the first two criteria [29].

## Results

Of all 35 participants included in our study at 12-month follow up, 31 responded on self-report inventories (88.6%), and 32 met for re-evaluation with the PSS-I (91.4%). The repeated measures ANOVA analyses found significant improvements in treatment outcomes across time for all measures. Effect sizes using partial eta squared values suggested large effects for all treatment outcomes at 12-month follow up, except for WSAS which showed a medium to large effect. When using Cohen’s *d*, large effects at 12-month follow up were observed especially for PTSD symptoms, while effects for depression, anxiety, interpersonal problems, and well-being showed moderate to large effects. A small to medium effect was observed for WSAS. The effects were very similar at 12-month follow up with those observed at post-treatment. The only exception was symptoms of PTSD, where further improvement after post-treatment was observed. When comparing post-treatment scores with 12-month follow-up, the improvement was comparable to a small effect size (PSS-I: *d*_*Post−12mFU*_ = 0.33, PCL-5: *d*_*Post−12mFU*_ = 0.21). When comparing 3-month scores with 12-month follow up, there were no clear changes (PSS-I: *d*_*3mFU − 12mFU*_ = -0.03, PCL-5: *d*_*3mFU − 12mFU*_ = -0.09). Thus, further improvement in post-traumatic symptoms occurred mainly during the first three months and then stabilized between 3- and 12-month follow-up. Table 2 summarizes the changes in outcome measures at all four points of assessment.

**Table 2:** Changes in symptoms, functioning, and well-being from pre-treatment to 12-month follow-up

	Pre	Post	3 m FU	12 m FU	F	p	η^2^ 12 m	d pre-post	d pre-12 m
PSS-I	32.07 (7.96)	17.92 (10.60)	14.43 (11.09)	14.71 (10.53)	42.92	< 0.001	0.558	1.51	1.86
PCL-5	51.89 (11.48)	34.07 (15.28)	29.60 (16.61)	30.87 (17.16)	29.54	< 0.001	0.465	1.32	1.44
BAI	26.14 (13.30)	16.81 (12.33)	16.74 (13.67)	16.64 (12.97)	20.89	< 0.001	0.381	0.72	0.72
BDI-II	28.38 (11.84)	19.30 (10.83)	18.51 (13.35)	19.38 (11.88)	11.95	< 0.001	0.260	0.80	0.76
WSAS	22.55 (7.96)	19.13 (7.87)	18.47 (9.08)	19.24 (9.30)	3.61	0.016	0.096	0.43	0.38
IIP-64	1.73 (0.47)	1.43 (0.50)	1.37 (0.57)	1.40 (0.41)	9.55	< 0.001	0.219	0.62	0.75
WHO-5	31.20 (20.54)	41.58 (18.20)	44.70 (17.77)	44.55 (17.73)	7.03	< 0.001	0.171	-0.53	-0.70

*Note*. Pre = pre-treatment, Post = post-treatment, 3 m FU = 3-month follow-up, 12 m FU = 12-month follow-up, PSS-I = PTSD Symptom Scale Interview, PCL-5 = PTSD Checklist for DSM-5, BAI = Beck Anxiety Inventory, BDI-II = Beck Depression Inventory II, WSAS = Work and Social Adjustment Scale, IIP-64 = Inventory of interpersonal problems, WHO-5 = World Health Organization Well-Being Index. *N* = 35. Cohen’s *d* calculated using pooled standard deviations

The treatment response rates showed that 60% of patients achieved recovery with respect to PTSD symptoms as measured with the PSS-I at 12-month follow up. When using the PCL-5 the rate was 45.7%. Recovery rates were lower for secondary outcome measures such as depression (26%), anxiety (23%), work- and social functioning (20%), and interpersonal problems (20%). For well-being, 49% were classified as recovered. Table 3 summarizes the treatment response rates.

**Table 3:** Treatment response rates at 12-month follow-up

		% (n)	
	Scoring below clinical cut-off	Achieving reliable change	CSC/recovery
PSS-I	62.9 (22)	82.9 (29)	60.0 (21)
PCL-5	48.6 (17)	65.7 (23)	45.7 (16)
BDI-II	37.1 (13)	51.4 (18)	25.7 (9)
BAI	42.9 (15)	48.6 (17)	22.9 (8)
WSAS	48.6 (17)	20.0 (7)	20.0 (7)
IIP-64	22.9 (8)	42.9 (15)	20.0 (7)
WHO-5	85.7 (30)	51.4 (18)	48.6 (17)

*Note*. CSC = clinically significant change (scoring below clinical cut-off and achieving reliable change). PSS-I = PTSD Symptom Scale – Interview for DSM-IV, PCL-5 = PTSD Checklist for DSM-5, BDI-II = Beck Depression Inventory, BAI = Beck Anxiety Inventory, WSAS = Work and Social Adjustment Scale, WHO-5 = The World Health Organisation – Five Well-Being Index, IIP-64 = Inventory of Interpersonal Problems. CSC/recovery involved both scoring below cut-off and achieving reliable change

According to PCL-5, 48.4% (*n* = 15) patients did not meet the criteria for a PTSD diagnosis at 12-month follow-up. In comparison, this rate was 51.7% at post-treatment, and 62.1% at 3-month follow-up. According to PSS-I, 43.8% (*n* = 14) did not meet criteria for a PTSD diagnosis at 12-month follow-up. In comparison this rate was 44.8% at post-treatment, and 51.7% at 3-month follow-up.

Eighteen participants did not receive any additional treatment during the 12-month follow-up period, while 17 received extra sessions (*M* = 12.3, Range = 1–26). Of these additional sessions 44% targeted relational or self-esteem issues, 34% further PTSD treatment, 9% depressive symptoms, 8% other anxiety symptoms, 3% various crises, and 2% discussions regarding further treatment. Three patients were referred to treatment for other presenting problems. When adding number of sessions given during the follow-up period as a covariate in the repeated measures analysis of PSS-I, it showed a significant interaction (*p* = .003, *η*^2^= 0.137), suggesting more symptoms in the group receiving additional sessions. Repeating the covariate analyses using the secondary outcome measures, showed a significant interaction with PCL-5 and WSAS, but not with BAI, BDI-II, IIP-64, or WHO-5.

## Discussion

The results supported the hypothesis: Patients receiving the intensive treatment maintained symptom reduction at 12-month follow-up. The gains were maintained on both main outcome measures of PTSD, and on secondary outcome measures. Improvement of posttraumatic symptoms occurred from pre-treatment to 3-month follow-up, and then stabilized. This study showed effect sizes from pre-treatment to 12-month follow-up of *d* = 1.44 for PCL-5 and *d* = 1.86 for PSS-I. In comparison, the meta-analysis encompassing 22 studies on LTFU of CBT for PTSD [6] found an effect size of 1.36. Other studies on long-term follow-up of 3-week intensive CBT [9] and 2-week PE [10] found effect sizes of 1.28 and 0.88. Furthermore, the present study had a high response rate at 12-month follow-up.

It is noteworthy to take into account that our effect sizes are on a sample of patients with previously unsuccessful treatment experiences or relapses. The high effect-sizes in this study may be attributed to the limited sample size and the study’s uncontrolled, non-randomized design. Additionally, properties of the treatment programme, such as therapist rotation, may have contributed to enhanced adherence to protocol.

This study further demonstrated large effect sizes for positively altering levels of depressive symptoms, anxiety, well-being, and interpersonal problems, This aligns with the studies conducted by Held and colleagues [9] and Yasinski and colleagues [10] who also reported large effect sizes on depression, supporting the notion that intensive treatment for PTSD can also yield positive impact on depressive symptoms. For work- and social functioning (WSAS) the effect size was lower (*d* = 0.38). Considering that the results showed a large effect on interpersonal functioning (IIP-64; *d* = 0.75), the smaller effect on WSAS might be more related to work capacity. A majority of the sample (*n* = 26, 74.3%) received long-term disability benefits or were on sick leave at treatment start, and only three of these patients had a part-time or full-time job at 12-month follow-up. Even though the intensive treatment influenced a broad range of symptoms, it did not seem to improve their occupational status after treatment. To achieve larger effects on WSAS, the treatment would likely need increased focus on functioning and work-related skills.

According to diagnostic status at 12-month follow-up, 44–48% (respectively PSS-I and PCL-5) of patients in the current study no longer met criteria for PTSD. Of all patients, 66–83% (respectively PCL-5 and PSS-I) reported reliable change. In the aforementioned 5–10 years LTFU study of patients that received individual CBT for PTSD, 78–82% did not meet the diagnostic criteria as measured with the Clinician Administered PTSD Scale [7]. At 9-month follow-up the same study population showed results similar to the current study with 50–55% not meeting the criteria for PTSD [8]. Even longer-term follow-up studies on intensive treatment for PTSD with controlled groups is therefore warranted to investigate whether similar delayed effects could be observed.

Similar to the current study, Resick and colleagues [7] found that additional treatment sessions in the follow-up period was associated with worse treatment outcome. This suggests that patients who respond well to the IOP did not require further treatment to maintain gains. Conversely, patients who initially responded inadequately to treatment continued to show limited improvement also after receiving additional sessions. There is a need for more research to understand why some patients respond poorly to treatment, and how to tailor treatment for this patient group.

There are different strengths and limitations that need to be considered when interpreting the results. The study population consisted of a clinical population who had previously received psychotherapy for PTSD without sufficient response. The high participation rate at 12-month follow-up enhances the generalizability. However, the sample size was small, and the absence of a control group and lack of randomization limit the ability to infer causality from the findings. In addition, the study employed PSS-I as clinical interview, complicating comparisons with studies utilizing the CAPS-5.

## Conclusions

IOP for PTSD applied to previous treatment non-responders, yields large positive outcomes that endure up to 12 months after treatment completion. Controlled trials are needed to establish relative efficacy. IOPs provide an additional treatment option for patients with PTSD, potentially facilitating quicker recovery, increased adherence to protocol, and prevention of session infrequency. Moreover, IOPs can enhance the accessibility of treatment for PTSD providing a concentrated treatment approach, suitable for patients with residency at a distance from treatment providers, or for those facing challenges in participating in conventional, weekly therapy sessions.

## Data Availability

The datasets used and analysed during the current study are available from the corresponding author on reasonable request.

## Abbreviations

PTSD: Posttraumatic stress disorder
EMDR: Eye Movement Desensitization and Reprocessing
PE: Prolonged Exposure
IOP: Intensive outpatient treatment
LTFU: Long-term follow up
CBT: Cognitive behavioural therapies
PA: Physical activity
RCI: Reliable change index
BDI-II: Beck Depression inventory
BAI: Beck Anxiety Inventory
WSAS: Work and Social Adjustment Scale
WHO-5: World Health Organization Well-Being Index
IIP-64: Inventory of Interpersonal Problems
PCL-5: PTSD Checklist for DSM-5
PSS-I: PTSD Symptom Scale Interview
CAPS-5: Clinician Administered PTSD Scale for DSM-5

## References

[1] Sherrill AM, Maples-Keller JL, Yasinski CW, Loucks LA, Rothbaum BO, Rauch SAM (2020). Perceived benefits and drawbacks of massed prolonged exposure: a qualitative thematic analysis of reactions from treatment completers. Psychol Trauma: Theory Res Pract Policy.

[2] Thoresen IH, Auren TJB, Klæth JR, Jensen AG, Engesæth C, Langvik EO (2022). Intensive outpatient treatment for PTSD: a thematic analysis of patient experience European. J Psychotraumatology.

[3] Gutner CA, Suvak MK, Sloan DM, Resick PA (2016). Does timing matter? Examining the impact of session timing on outcome. J Consult Clin Psychol.

[4] Sciarrino NA, Warnecke AJ, Teng EJ. A systematic review of intensive empirically supported treatments for posttraumatic stress disorder. 2020;33(4):443–54. 10.1002/jts.22556 32598561

[5] Hoppen TH, Kip A, Morina N (2023). Are psychological interventions for adult PTSD more efficacious and acceptable when treatment is delivered in higher frequency? A meta-analysis of randomized controlled trials. J Anxiety Disord.

[6] Weber M, Schumacher S, Hannig W, Barth J, Lotzin A, Schäfer I (2021). Long-term outcomes of psychological treatment for posttraumatic stress disorder: a systematic review and meta-analysis. Psychol Med.

[7] Resick PA, Williams LF, Suvak MK, Monson CM, Gradus JL (2012). Long-term outcomes of cognitive–behavioral treatments for posttraumatic stress disorder among female rape survivors. J Consult Clin Psychol.

[8] Resick PA, Nishith P, Weaver TL, Astin MC, Feuer CA (2002). A comparison of cognitive-processing therapy with prolonged exposure and a waiting condition for the treatment of chronic posttraumatic stress disorder in female rape victims. J Consult Clin Psychol.

[9] Held P, Zalta AK, Smith DL, Bagley JM, Steigerwald VL, Boley RA (2020). Maintenance of treatment gains up to 12-months following a three-week cognitive processing therapy-based intensive PTSD treatment programme for veterans. Eur J Psychotraumatology.

[10] Yasinski CW, Watkins LE, Maples-Keller JL, Ragsdale KA, Sherrill AM, Burton MS (2022). Long-term effectiveness of a prolonged exposure-based intensive outpatient program for veterans with posttraumatic stress disorder. J Psychiatr Res.

[11] Hurley EC (2018). Effective treatment of veterans with PTSD: comparison between intensive daily and weekly EMDR approaches. Front Psychol.

[12] Auren TJB, Jensen AG, Klæth JR, Maksic E, Solem S (2021). Intensive outpatient treatment for PTSD: a pilot feasibility study combining prolonged exposure therapy, EMDR, physical activity, and psychoeducation. Eur J Psychotraumatology.

[13] van Woudenberg C, Voorendonk EM, Bongaerts H, Zoet HA, Verhagen M, Lee CW (2018). Effectiveness of an intensive treatment programme combining prolonged exposure and eye movement desensitization and reprocessing for severe post-traumatic stress disorder. Eur J Psychotraumatology.

[14] Auren TJB, Klæth JR, Jensen AG, Solem S (2022). Intensive outpatient treatment for PTSD: an open trial combining prolonged exposure therapy, EMDR, and physical activity. Eur J Psychotraumatology.

[15] Sheehan DV, Lecrubier Y, Sheehan KH, Amorim P, Janavs J, Weiller E (1998). The mini-international neuropsychiatric interview (M.I.N.I): the development and validation of a structured diagnostic psychiatric interview for DSM-IV and ICD-10. J Clin Psychiatry.

[16] Brown T, Di Nardo P, Barlow D (1994). Anxiety disorders interview schedule for DSM-IV (ADIS-IV).

[17] Foa EB, Riggs DS, Dancu CV, Rothbaum BO (1993). Reliability and validity of a brief instrument for assessing post-traumatic stress disorder. J Trauma Stress.

[18] Foa EB, Hembree EA, Rothbaum BO (2007). Treatments that work. Prolonged exposure therapy for PTSD: emotional processing of traumatic experiences: therapist guide.

[19] Shapiro F, Korn DL, Stickgold R, Maxfield L, Smyth NJ. Eye Movement Desensitization and Reprocessing (EMDR) Therapy. 3rd ed. Guilford Press; 2018.

[20] Blevins CA, Weathers FW, Davis MT, Witte TK, Domino JL (2015). The posttraumatic stress disorder checklist for DSM-5 (PCL‐5): development and initial psychometric evaluation. J Trauma Stress.

[21] The Improving Access to Psychological Therapies Manual. Appendices and helpful resources 2018 [Available from: https://www.england.nhs.uk/wp-content/uploads/2018/06/iapt-manual-resources-v2.pdf.

[22] Beck AT, Steer RA, Brown GK (1996). Manual for the Beck depression inventory-II.

[23] Beck AT, Epstein N, Brown G, Steer RA (1988). An inventory for measuring clinical anxiety: psychometric properties. J Consulting Clin Psychol.

[24] Mundt JC, Marks IM, Shear MK, Greist JM (2002). The work and Social Adjustment Scale: a simple measure of impairment in functioning. Br J Psychiatry.

[25] Topp CW, Østergaard SD, Søndergaard S, Bech P (2015). The WHO-5 well-being index: a systematic review of the literature. Psychoterapy Psychosom.

[26] Löwe B, Spitzer RL, Gräfe K, Kroenke K, Quenter A, Zipfel S (2004). Comparative validity of three screening questionnaires for DSM-IV depressive disorders and physicians’ diagnoses. J Affect Disord.

[27] Alden LE, Wiggins JS, Pincus AL (1990). Construction of circumplex scales for the inventory of interpersonal problems. J Pers Assess.

[28] Schafer J (1997). Analysis of incomplete Multivariate Data.

[29] Jacobson NS, Truax P (1991). Clinical significance: a statistical approach to defining meaningful change in psychotherapy research. J Consult Clin Psychol.

